# Infection Rates and Characterisation of *Rickettsia africae* (Rickettsiaceae) Detected in *Amblyomma* Species from Southern Africa

**DOI:** 10.3390/microorganisms12081663

**Published:** 2024-08-13

**Authors:** Andeliza Smit, Fernando C. Mulandane, Stephané H. Wójcik, Choolwe Malabwa, Gourgelia Sili, Stephen Mandara, Hannah Rose Vineer, Zinathi Dlamkile, Wilhelm H. Stoltsz, Darshana Morar-Leather, Benjamin L. Makepeace, Luis Neves

**Affiliations:** 1 Ticks Research Group, Department of Veterinary Tropical Diseases, Faculty of Veterinary Science, University of Pretoria, Onderstepoort 0110, South Africa; stephaneheike@gmail.com (S.H.W.); zinathi.lukanji@up.ac.za (Z.D.); hein.stoltsz@up.ac.za (W.H.S.); darshana.morar@up.ac.za (D.M.-L.); luis.neves@up.ac.za (L.N.); 2 Biotechnology Centre, Eduardo Mondlane University, Maputo 1102, Mozambique; fernandomulandane@gmail.com; 3 Central Veterinary Research Institute, Lusaka P.O. Box 33980, Zambia; choolwemalabwa@gmail.com; 4 Department of Basic Science, Faculty of Veterinary Medicine, University Jose Eduardo dos Santos, Huambo P.O. Box 2458, Angola; gourgeliasili@hotmail.com; 5 Department of Animal Production Sciences, Marondera University of Agricultural Sciences and Technology, Marondera P.O. Box 35, Zimbabwe; stevemandara@gmail.com; 6 Department of Infection Biology and Microbiomes, Institute of Infection, Veterinary & Ecological Sciences, University of Liverpool, Liverpool L69 7ZX, UK; hannah.vineer@liverpool.ac.uk

**Keywords:** African tick bite fever, tick-borne diseases, prevalence, zoonotic disease, tick-borne pathogen, wildlife

## Abstract

Tick-borne rickettsioses are considered among the oldest known vector-borne zoonotic diseases. Among the rickettsiae, *Rickettsia africae* is the most reported and important in Africa, as it is the aetiological agent of African tick bite fever (ATBF). Studies describing the prevalence of *R*. *africae* in southern Africa are fragmented, as they are limited to small geographical areas and focused on *Amblyomma hebraeum* and *Amblyomma variegatum* as vectors. *Amblyomma* spp. ticks were collected in Angola, Mozambique, South Africa, Zambia and Zimbabwe during the sampling period from March 2020 to September 2022. *Rickettsia africae* was detected using the *ompA* gene, while characterisation was conducted using *omp*, *ompA*, *ompB* and *gltA* genes. In total, 7734 *Amblyomma* spp. ticks were collected and were morphologically and molecularly identified as *Amblyomma eburneum*, *A*. *hebraeum*, *Amblyomma pomposum* and *A*. *variegatum*. Low levels of variability were observed in the phylogenetic analysis of the *R. africae* concatenated genes. The prevalence of *R*. *africae* ranged from 11.7% in South Africa to 35.7% in Zambia. This is one of the largest studies on *R*. *africae* prevalence in southern Africa and highlights the need for the inclusion of ATBF as a differential diagnosis when inhabitants and travellers present with flu-like symptoms in the documented countries.

## 1. Introduction

*Rickettsia* species are highly fastidious, obligate intracellular Gram-negative bacteria classified in the family Rickettsiaceae [1]. Tick-borne rickettsioses are considered among the oldest known vector-borne zoonotic diseases [2]. Most pathogenic species of *Rickettsia* are categorised into three groups, namely the typhus group (TG), transitional group (TRG), and the spotted fever group (SFG), while the ancestral group (AG) is still under debate [3]. The SFG contain 23 species, of which 16 are pathogenic, including *Rickettsia conorii,* the causative agent of Mediterranean spotted fever [4], *R. conorii* subsp. *raoultii* and *Rickettsia slovaca,* which causes rickettsial lymphadenopathy [5] and *Rickettsia rickettsii,* which causes rocky mountain spotted fever [6]; all of which are associated with ticks as the main vectors. By contrast, the TG contains only two species, *Rickettsia prowazekii* and *Rickettsia typhi*, which are associated with lice or fleas as their vectors, respectively [7,8,9]. Finally, the TRG contains both arthropod-restricted symbionts and human pathogens transmitted by fleas, ticks and mites [10,11]. Among the rickettsiae, *Rickettsia africae* is the most frequently reported and significant in Africa, as it is the aetiological agent of African tick bite fever (ATBF) [12].

The symptoms of ATBF resemble those of malaria. It is an acute, flu-like illness with symptoms comprising fever, myalgia, severe headache, nausea and fatigue. In most cases, an inoculation eschar is present, though it may go unnoticed on darker skin tones, with regional lymphadenitis, cutaneous rash and, in rare cases, aphthous stomatitis [13]. Whereas flu-like symptoms are common, some cases may present with some but not all ailments, while other cases may be asymptomatic. Severe disease is rare, with no records of life-threatening complications or deaths associated with the disease.

**Figure 1:**
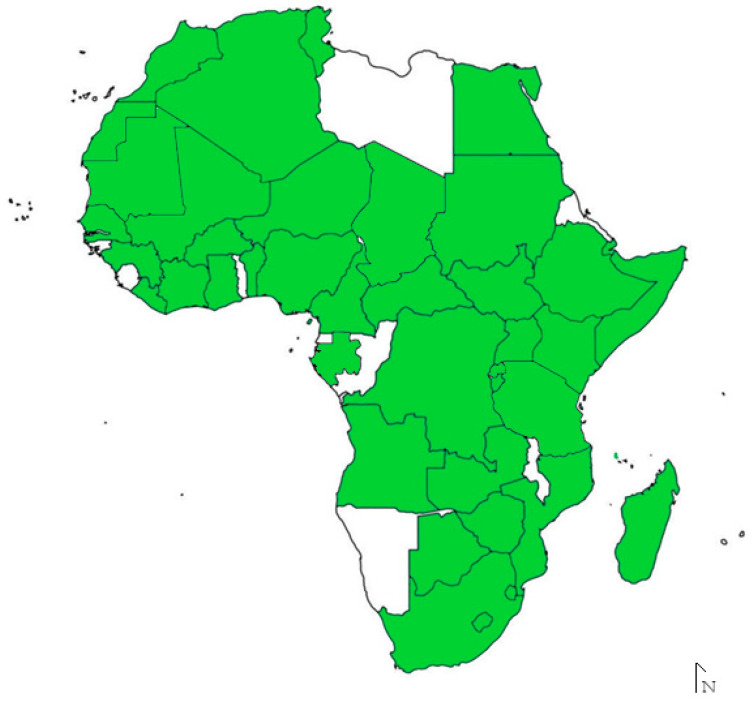
Distribution of *Rickettsia africae* in Africa based on detection in either vector ticks or human hosts. White shaded areas indicate countries where *R*. *africae* has not been detected or investigated. Image was constructed with information obtained from Genbank, Mazhetese, et al. [21], Pillay, et al. [24] and Zhang, et al. [25].

*Amblyomma* spp. ticks are known to be the primary vectors of *R. africae* and hence the distribution of *R. africae* is reliant on the distribution of the tick vectors [13] (Figure 1). Therefore, the seasonality of *R*. *africae* is also dependent on the seasonality of the vectors [14,15]. *Amblyomma variegatum* (Fabricius, 1794) [16] is the most widespread vector, found in central, western and eastern Africa as well as the West Indies; its New World distribution comprises the islands of Guadeloupe, Martinique, St. Kitts and Nevis, and Antigua following introductions from Africa [7]. Accordingly, *R. africae* is largely restricted to the African continent [17,18,19], although the disease it causes, ATBF, has been detected in many countries globally due to increases in tourism to sub-Saharan Africa and *R. africae* dispersal via the tick vector on livestock or wild hosts [20,21]. In endemic areas, the infection rate of ticks with *R*. *africae* can reach up to 100%, and among travellers, ATBF is the second most commonly identified cause of systemic febrile illness, following malaria [22,23].

Several gaps are evident in the literature focusing on *R*. *africae*, including a lack of information on the role of mammalian hosts in the maintenance of *R*. *africae* and the prevalence rates of infection between male and female ticks and its effects on pathogen transmission. Regarding the role of the mammalian hosts, conflicting hypotheses on the natural reservoirs for *R*. *africae* can be found in the literature, as data on the role of mammalian hosts in the maintenance of *R*. *africae* is scant and contradictory. Kelly, et al. [26] reported that 26–90% of cattle in Zimbabwe had antibodies against *R*. *africae,* although none displayed any clinical symptoms. During their study, they isolated *Rickettsia*-like organisms from *Amblyomma hebraeum* ticks and cultured these isolates. Eight naïve cattle and three male guinea pig subjects were inoculated with these isolates. Kelly, et al. [26] observed seroconversion in all eight of the naïve cattle breeds and all three of the guinea pig subjects, suggestive of the possible maintenance of *R*. *africae* in cattle. It is key to note that the conclusion of Kelly, et al. [26] on the maintenance of *R*. *africae* in cattle was based solely on the seroconversion of naïve cattle. It has also been reported that *R*. *africae* serological assays have low specificity [20]. To date, no studies have been conducted using DNA methods to determine whether cattle play a role in the maintenance of *R*. *africae*. The other possibility is that *Amblyomma* spp. does not require a mammalian reservoir. Although *Amblyomma* spp. are well documented to transmit *R*. *africae* transovarially, no studies are currently available on the efficiency of the transovarial transmission across several tick generations [21]. Thus, insufficient evidence is available on the maintenance of *R. africae* in the field.

Knowledge on the prevalence rate of *R*. *africae* in male and female ticks could contribute to the disease epidemiology of *R*. *africae*, which has been mostly absent in the literature to date. Male ticks are more likely to stay attached to hosts when compared to female ticks; however, males have been documented to detach from host during nights, changing predilection site or even hosts [27]. Both male and female *Amblyomma* spp. are known to be aggressive hunters with high biting ratios. The biting ratio alongside the attachment duration could impact pathogen transmission. Therefore, documenting and understanding the difference in infection rate between male and female ticks are crucial for a comprehensive understanding of *R*. *africae* transmission and epidemiology.

Despite the number of reports that have detected *R*. *africae* in several vectors, studies describing the prevalence of *R*. *africae* in southern Africa are fragmented, limited to small geographical areas and focused on the primary vectors only (*Amblyomma hebraeum* (Koch, 1844) and *A*. *variegatum*) [28,29,30,31,32,33,34,35]. Recent studies list 21 *Amblyomma* spp. occurring in south-eastern Africa, most of which have limited to no information on infection rates with *R*. *africae*. The paucity of information on prevalence rates is compounded by a lack of data on *R*. *africae* strains from these neglected vectors.

The characterisation and systematics of *R*. *africae* has relied on the use of several molecular markers. Common markers include the outer-membrane protein OmpA (*ompA*) [1], outer-membrane protein OmpB (*ompB*) [36] and citrate synthase (*gltA*) [37]. Less common markers encompass the 17 kDa surface antigen (*omp*) [38], surface cell antigen 4 (*sca4*) [39], 16S rRNA [40] and the internal transcribed spacer (ITS) [41]. Rarely used markers include dksA-xerC, mppA-purC and rpmE-tRNAfMet, which are all intergenic spacers [4]. However, it is standard practice to use a combination of several of the above-mentioned markers [24]. In recent years, the popularity of whole genome sequencing has increased due to its lower cost, yet the first and only whole genome sequence of *R*. *africae* became available in 2009 [42]. Rickettsiae are known for their high level of collinearity, with the main markers exhibiting homogeneity ranging from 82.2% to 99.8%, which hinders intra-species differentiation [43].

Although intraspecific variability of SFG rickettsiae is generally low [43], a greater level of heterogeneity has been reported when using the *ompA* and *ompB* molecular markers as compared to the intergenic spacers [30,44]. Intra-species variation was more prominent in studies that have applied three or more markers [45,46,47]. In this study, we investigated the prevalence of *R*. *africae* in *Amblyomma* spp. ticks collected in southern Africa and characterised phylogenetic relationships of *R*. *africae* using the *ompA*, *ompB*, *gltA* and *omp* genetic markers.

## 2. Materials and Methods

### 2.1. Sample Collection and Identification

**Figure 2:**
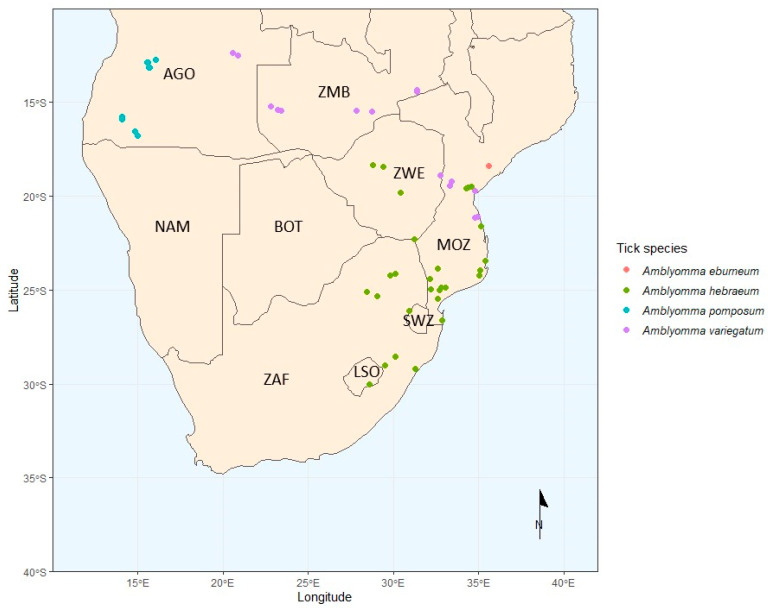
Map of southern Africa, illustrating the *Amblyomma* spp. collection points during the 2020–2022 sampling period performed by Smit et al. [41]. The colour of the dots represents the species of *Amblyomma* spp. ticks collected at the respective sampling points. Country codes: AGO—Angola, BOT—Botswana, LSO—Lesotho, MOZ—Mozambique, NAM—Namibia, SWZ—Eswatini, ZAF—South Africa, ZMB—Zambia, and ZWE—Zimbabwe.

*Amblyomma* spp. ticks from Smit, et al. [48] were used for this study. These were collected in Angola, Zambia and Zimbabwe, from randomly sampled cattle and goats, while in Mozambique, *Amblyomma* spp. were collected from both cattle and legally hunted wildlife (Figure 2). Samples were also used from Dlamkile, et al. [49], who collected *Amblyomma* spp. ticks from randomly sampled cattle in South Africa from 2020 to 2022.

Morphological identification of the species level was conducted using identification keys from Walker et al. [50] and Voltzit and Keirans [51], while morphological characteristics were documented in Smit, et al. [48].

### 2.2. DNA Extraction and Amplification

DNA was extracted from halved individual ticks using the Chelex 100 resin method as described in Smit, et al. [52]. Tick identification was confirmed in Smit, et al. [48] by molecular characterisation targeting the 12S rRNA [53], 16S rRNA [54], cytochrome oxidase I (*coi*) [55] and cytochrome B (*cytB*) [56] molecular markers.

With the use of Cannon and Roe [57], the sample size for the tick collection in Mozambique was calculated as the number of ticks required to detect at least one infected tick, at a confidence level of 95%, assuming an infection rate of 10% and an infinite population. This resulted in 132 ticks required to provide statistically significant results. A subsample of 160 ticks per species was screened for *R*. *africae* using the *ompA* gene as described by Mazhetese, et al. [58]. 

*Rickettsia africae* characterisation was conducted by amplifying four gene regions: *omp*, *ompA*, *ompB* and *gltA* (Table 1). In total, 50 *Amblyomma* ticks positive for *R*. *africae* were selected to ensure five positive ticks per species per country. Each gene amplification consisted of a 25 µL reaction comprising 10 µL Phusion Flash PCR Master Mix (1X Final concentration), 0.5 µL each of the corresponding primers (Table 1) (final concentration of 0.5 µM per primer), 7 µL double-distilled water and 2 µL sample DNA. The PCR cycling conditions for *omp, ompB* and *gltA* were as follows: initial denaturation at 98 °C for 10 s, followed by 35 cycles of 92 °C for 1 s, 50 °C for 5 s, and 68 °C for 15 s. Final annealing was conducted at 72 °C for 2 min with a hold stage at 4 °C. The *ompA* cycling conditions were an initial denaturation step at 98 °C for 10 s, followed by 30 cycles of 92 °C for 1 s, 51 °C for 5 s, and 68 °C for 15 s. Final annealing was conducted at 70 °C for 1 min with a hold stage at 4 °C. A sample from a previous study which was proven to be *R. africae* by sequencing was used as a positive control for each amplification. A no-template (double-distilled water) negative control was included in each amplification.

PCR products were separated on a 1.5% agarose gel to confirm whether a sample tested positive for *R*. *africae* and that the band was the expected size to be sent for sequencing. The gel was visualised using the Bio-Rad gel documentation system with assisted visualisation programming. Samples that produced visible single bands were sent to the Central Analytical Facility (CAF), Stellenbosch, South Africa, for Sanger sequencing in both directions.

### 2.3. Phylogenetic Analysis

Contigs of forward and reverse sequences were constructed using CLC main workbench version 23.0.2 (developed by CLC Bio, http://www.clcbio.com (accessed on 1 August 2024)). These contigs of each individual sample for each individual gene were manually corrected on CLC main workbench version 23.0.2. Manual corrections consisted of base calling, where base inconsistency between the forward and reverse sequences were corrected. For *R*. *africae* characterisation, individual genes and a concatenated matrix were used for processing. Reference sequences were obtained from the GenBank database and used for comparison (https://www.ncbi.nlm.nih.gov/genbank/) (accessed on 17 February 2024) (Supplementary Table S1). For each of the individual genes (*omp*, *ompA*, *ompB* and *gltA*), *R. conorii* was used as the outgroup. For the concatenated matrix, *R. conorii* subsp. *raoultii* was used as an outgroup since it was one of the few *Rickettsia* strains for which all genes were available from the same sample on GenBank. Orientation of the assembled contigs was evaluated, and these contigs were aligned alongside the reference sequences with the use of the online version of MAFFT version 7 (developed by http://mafft.cbrc.jp/alignment/server/index.html) (accessed on 17 February 2024) with default parameters. The aligned matrix was manually viewed, edited and truncated using MEGA 11. The best-fit model was determined using the jModelTest2 [59] on the CIPRES Science Gateway (https://www.phylo.org/ (accessed on 1 August 2024)) platform. The file format was changed depending on program requirements, using FaBox version 1.61 (https://users-birc.au.dk/palle/php/fabox/index.php) (accessed on 17 February 2024). Bayesian analysis was performed in MrBayes version 3 [60]. The Hasegawa–Kishino-Yano (HKY) model with gamma rates and invariable sites was used for the concatenated *R*. *africae* tree. Individual gene tree models are listed in the figure descriptions. Five Monte Carlo Markov Chains (MCMC) were run for 5,000,000 iterations, saving every 1000th tree, while the first 25% of drafted trees were discarded. Tracer version 1.6 [61] was used for the inspection of estimated sample size (ESS) (>200) and parameter sampling using graphical plots indicating parameter stabilisation. The resulting tree was visualised and edited in iTOL version 6.8 [62].

### 2.4. Statistical Analysis

The statistical significance of *R. africae* prevalence between the males and females of each species was calculated using the numbers collected for each respective sex, and the number of positives for each respective sex. The statistical significance of *R. africae* prevalence between the *Amblyomma* spp. in each country was calculated using the total number collected in a specific country and the total number of positives for each species. A χ^2^ analysis was conducted in Microsoft Excel version 2402 to evaluate the independence between infection rate and sex of tick species, as well as to evaluate the independence between infection rate and tick species. If the expected number was lower than five, then the Fisher’s exact test was used instead. A Bonferroni correction was applied, resulting in a *p*-value threshold for acceptance of 0.004 (0.05/12 tests).

### 2.5. Ethical Considerations

This study obtained approval from the Research and Animal Ethics Committee of the University of Pretoria (REC 121-20). Furthermore, consent was granted by the Department of Agriculture, Land Reform, and Rural Development (DALRRD) in South Africa, under Section 20 of the Animal Diseases Act 1984 (Act no. 35 of 84) (12/11/1/1 (1937SS)).

## 3. Results

In total, 7734 adult *Amblyomma* ticks were collected in southern Africa and were morphologically identified as *Amblyomma eburneum* (Gerstäcker, 1873) [63] (*n* = 208), *A*. *hebraeum* (*n* = 4758), *Amblyomma pomposum* (Dönitz, 1909) [64] (*n* = 191) and *A*. *variegatum* (*n* = 2577) [48].

Amplification success varied amongst the genes; *ompA* had a success rate of 78% (39/50), while *ompB* had a success rate of 86% (43/50), *omp* had a success rate of 74% (37/50), and *gltA* had a success rate of 82% (41/50). Although 37 samples did amplify using the *omp* gene, only 29 produced high-quality sequences, while the remaining nine were of low quality and could not produce contigs. The *gltA* locus also experienced low sequence quality, where 41 samples amplified but only 36 were of good quality and formed contigs. All samples that produced visible single bands for *ompA* and *ompB* generated sequences that were of high quality, all of which formed contigs. All sequences were deposited in GenBank (Supplementary Table S1).

Individual gene trees are located in Supplementary Data S2. The Bayesian analysis of the *ompA* gene illustrated a clear clustering of all the sequences obtained in this study with those obtained from GenBank, with the exception of three *R*. *africae* sequences obtained from *Hyalomma* spp., which formed their own clade (probability of 0.98) (Supplementary Data S2: Figure S1). The *ompB* analysis depicted the majority of the sequences from this study clustering together with reference sequences from GenBank (Supplementary Data S2: Figure S2). Nine sequences were observed branching from the main clade with a high probability (0.98). Of these nine sequences, seven were collected from wildlife (*Syncerus caffer*) in Mozambique; the other two sequences were derived from *A*. *variegatum* collected from livestock (*Bos taurus*) in Angola and Mozambique. The Bayesian analysis of the *omp* gene clustered all the sequences of this study alongside references obtained from GenBank (Supplementary Data S2: Figure S3). Four branches were observed extending from the main clade; however, three of these nodes were not supported (probability lower than 0.95). Two of these separations are located on long branches; when the sequences were compared to those on GenBank, identities of 96% or higher were observed with *R*. *africae* 17 kDa sequences. The *gltA* topology displayed greater variation (Supplementary Data S2: Figure S4). Three separate clades were observed for *R*. *africae*; the first contained two *R*. *africae* sequences from *Hyalomma* and one sequence obtained from *Gorilla gorilla* (gorilla), the second clade contained one *R*. *africae* sequence from *Amblyomma compressum*, and the last clade contains all the sequences from this study and several reference sequences from GenBank. Two long branches were observed separating from the main clade; however, these nodes were not supported (probability lower than 0.95). 

**Figure 3:**
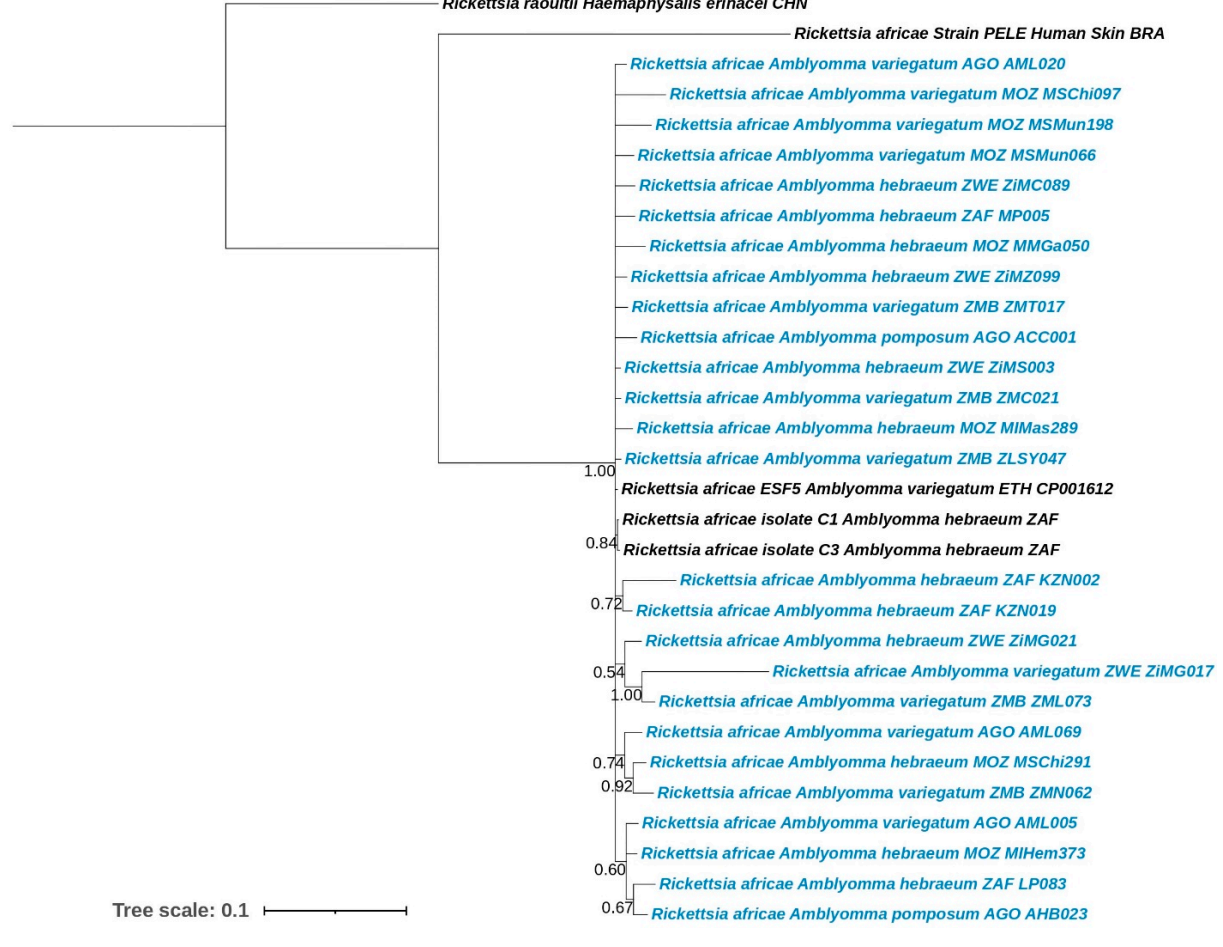
Bayesian inference analysis of the concatenated *Rickettsia africae* genes (*omp*, *ompA*, *ompB* and *gltA*). Analysis was performed using the HKY model with gamma rates and invariable sites. Posterior probability values are indicated at each branch node. Sequences from this study are in blue. Country of origin is indicated in the sample ID as: AGO—Angola, BRA—Brazil, CHN—China, ETH—Ethiopia, MOZ—Mozambique, ZAF—South Africa, ZMB—Zambia, and ZWE—Zimbabwe.

The Bayesian analysis of the phylogeny based on the concatenated genes indicated very little variation between the sequences obtained from this study (Figure 3). The majority of the samples from this study clustered with reference sequences from Pillay and Mukaratirwa [47] (C1 and C3) from South Africa, and with the *R*. *africae* whole genome sequence from an *A*. *variegatum* tick from Ethiopia. Variation in the *R*. *africae* sequences was observed for isolates obtained from humans. Several *R. africae* samples branched separately from the main clade; however, these nodes were not supported (probability lower than 0.95).

Our results from the *R*. *africae* conventional PCR showed that the prevalence in Angola was 24.4% (108/443) (Supplementary Table S2). *Amblyomma pomposum* had a prevalence of 41.9% (80/191), of which 25% of females (1/4) and 42.2% of males (79/187) tested positive. The *A*. *variegatum* from Angola had a prevalence of 11.1% (28/252), of which 25% of females (4/16) and 10.2% of males (24/236) tested positive. There was no association between *R*. *africae* infection rates and *A*. *pomposum* sex (*p* = 0.087), or between *R*. *africae* infection rates and *A*. *variegatum* sex (*p* = 0.641). However, there was an association between *R. africae* infection and tick species in Angola (X^2^_(1, *n* = 443)_ = 55.813, *p* < 0.001), with higher infection rates observed in *A. pomposum* (Supplementary Table S2).

In Mozambique, the overall prevalence of *R*. *africae* was 25.7% (301/1170), of which the prevalence in ticks collected from livestock was 28.6% (244/854) and in ticks collected from wildlife, 18.0% (57/316) (Supplementary Table S2). In livestock, two *Amblyomma* spp. were identified in Mozambique, *A*. *hebraeum* and *A*. *variegatum*. The prevalence of *R*. *africae* in *A*. *hebraeum* from livestock was 33.5% (181/541), of which 19.3% (33/171) of females and 40% (148/370) of males tested positive; this difference was statistically significant (X^2^_(1, *n* = 541)_ = 22.513, *p* < 0.001). The prevalence of *R*. *africae* in *A*. *variegatum* from livestock was 20.1% (63/313), of which 17.9% (14/78) of females and 20.9% (49/235) of males tested positive; this difference was not significant (X^2^_(1, *n* = 313)_ = 0.307, *p* = 0.58). However, there was a statistical association between *R. africae* infection rate and the two tick species found on livestock from Mozambique (X^2^_(1, *n* = 854)_ = 17.261, *p* = 0.002). 

In the wildlife from Mozambique, two *Amblyomma* species were identified as *A*. *eburneum* and *A*. *variegatum*. The prevalence of *R*. *africae* in *A*. *eburneum* from wildlife was 14.1% (49/235), of which 19.5% (8/41) of females and 12.2% (14/115) of males tested positive; this difference was not significant (*p* = 0.472). The prevalence of *R*. *africae* in *A*. *variegatum* from wildlife was 21.9% (35/160), of which 15.1% (8/53) of females and 25.2% (27/107) of males tested positive; this difference was also not significant (X^2^_(1, *n* = 160)_ = 2.132, *p* = 0.144). There was no statistical association between *R. africae* infection rates and the two tick species in wildlife from Mozambique (X^2^_(1, *n* = 316)_ = 3.226, *p* = 0.072); nor was there a significant association in the *R*. *africae* prevalence between livestock *Amblyomma* spp. and wildlife *Amblyomma* spp. (X^2^_(1, *n* = 1170)_ = 13,393, *p* = 0.004).

The infection rates by sex of the *A. hebraeum* from South Africa were not documented in the previous study [49] and thus no comparison can be made between the sexes for this country. The overall prevalence of *R*. *africae* was 11.4% (48/421) (Supplementary Table S2). 

The overall *R*. *africae* prevalence in *A. variegatum* from Zambia was 35.7% (202/566) (Supplementary Table S2). Females had a prevalence of 37.5% (12/32) and males had a prevalence of 35.6% (190/534); this difference was not statistically significant (X^2^_(1, *n* = 566)_ = 0.048, *p* = 0.826).

The overall *R*. *africae* prevalence from the ticks collected in Zimbabwe was 28.7% (99/345) (Supplementary Table S2). Both *A*. *hebraeum* and *A*. *variegatum* were collected in Zimbabwe. The prevalence in *A*. *hebraeum* was 29.3% (98/334), where 29% (38/131) of females tested positive and 29.6% (60/203) of males tested positive (X^2^_(1, *n* = 334)_ = 0.012, *p* = 0.914). Too few *A*. *variegatum* (11 in total) were collected in Zimbabwe for a valid statistical analysis by sex. No statistically significant differences in the *R*. *africae* prevalence between the species were observed in Zimbabwe (*p* = 0.189).

## 4. Discussion

In this study, we investigated the prevalence of *R*. *africae* in *Amblyomma* ticks from southern Africa. Overall, four *Amblyomma* spp. were studied: *A*. *eburneum*, *A*. *hebraeum*, *A*. *pomposum* and *A*. *variegatum*. Based on molecular findings, insufficient evidence is available to differentiate *A*. *pomposum* from *A*. *variegatum* [48]. However, based on morphological descriptions and behavioural features, Smit, et al. [48] still regarded *A*. *pomposum* and *A*. *variegatum* as separate species, which requires further investigation. Since the taxonomy of the genus *Amblyomma* has remained unchanged, *A*. *pomposum* and *A*. *variegatum* are regarded as distinct species in this manuscript.

The phylogenetic analysis of the *R*. *africae ompA* gene indicated homogeneity, except for three sequences obtained from *Hyalomma* in previous studies [65,66], branching from the main clade with high probability. This separation observed between *R*. *africae* obtained from *Amblyomma* spp. and *Hyalomma* spp. has been documented in several articles such as Pillay and Mukaratirwa [47], Pillay, et al. [67] and Thekisoe, et al. [68]. This highlights that the variability of *R*. *africae* depends on tick origin. It should be noted that the *R*. *africae* vector competency of *Hyalomma* spp. has not been validated; thus, the sequences of *R*. *africae* isolates from *Hyalomma* spp. may represent residual DNA from co-feeding with other tick species or from an infected blood meal. The *ompB* analysis depicted a clear separation between the majority of the sequences and nine sequences generated during this study (probability of 0.98), of which eight were from Mozambique and one from Angola. Of these nine sequences that branched from the main clade, seven were collected from wildlife. Few reference sequences could be used for the *ompB* analysis due to several large gaps observed in the alignment resulting in inaccurate topologies. Pillay and Mukaratirwa [47], from which the primer sequences were obtained, noted that *ompB* alongside *gltA* provided the best intraspecific resolution. This contradicts what we observed for the *ompB* analysis where all the sequences, aside from the nine that formed a distinct branch, cluster together regardless of vector species or geographic location. We did, however, observe high levels of resolution using the *gltA* and 17 kDA genes, with clear separation between *R*. *africae* from different vectors such as *Hyalomma*, *A*. *compressum* and the *Amblyomma* spp. from this study. No separation based on geographical location was observed in any of the individual gene topologies.

Phylogenetical analysis of the *R*. *africae* concatenated sequences from this study (*omp*, *ompA*, *ompB* and *gltA*) illustrated low levels of variability, in which most of the *R*. *africae* sequences from *A*. *pomposum*, *A. hebraeum* and *A*. *variegatum* clustered together regardless of the country or type of host. This is to be expected due to the high level of collinearity within members of the SFG [9]. The homogeneity in *R*. *africae* observed in our study was also reported by Kimita, et al. [45], Iweriebor, et al. [44] and Pillay, et al. [67]. Kimita, et al. [45] highlighted the greater resolution obtained from the concatenation of these genes as compared to using each gene separately during phylogenetic analysis. Several sequences from this study were excluded from the concatenated analysis, since the samples did not have all four genes available for each isolate or clone (Supplementary Table S1). There were limited sequences to which we could compare since few are available for the *omp* locus on GenBank. Several other studies have also reported problematic amplification of the abovementioned loci, which could be due to variation in primer binding sites or sensitivity of the assays to inhibitors [44]. This high level of collinearity in *R*. *africae* provides a stable framework for vaccine development. However, most of the current studies focus solely on structural genes, such as the ones used in this study. To advance vaccine development, research should explore functional and phenotypic markers, as well as their expression levels, associated with virulence, cell invasion and pathogen maintenance. 

The role of mammalian hosts in the maintenance of *R*. *africae* is still disputed with no molecular evidence in support of mammalian infection, as opposed to exposure measured by serology [21]. Although, multiple pathogens are known to be acquired by *Amblyomma* spp. ticks during ingestion of a blood meal from an infected host, such as *Ehrlichia ruminantium* [69] and *Theileria mutans* [70], several studies have determined a low prevalence in the cattle but a high prevalence in attached ticks. Diobo, et al. [71] conducted a study in Côte d’Ivoire, where they collected ticks and blood samples from cattle in five areas. Screening was performed of both the ticks and blood samples using the poT15-dam2 gene of *R*. *africae.* Diobo, et al. [71] noted a 43.67% positivity rate for *R*. *africae* in ticks collected from cattle, while no positives were detected in blood samples. Barradas, et al. [31] reported similar results during their study in Huambo, Angola. From the 98 blood samples they collected from cattle, none tested positive for *R*. *africae* using the *ompB* gene region. From the 116 ticks collected, 5 (4.3%) tested positive for *R*. *africae*. These findings support the hypothesis that mammalian hosts are not reservoirs for *R*. *africae.* If cattle were the reservoirs, one would expect to see high infection rates in their blood when detecting high infection rates in the ticks. However, current documentation appears to support that cattle become infected only after infected ticks feed on them. Ultimately, the role of mammals as reservoirs for *R*. *africae* should be investigated. Nevertheless, with no experimental evidence, we cannot fully exclude the possibility that the *R*. *africae* detected in the ticks in this study originated from the bloodmeal.

If mammalian hosts are not reservoirs for *R*. *africae*, we can then speculate that *R*. *africae* is a vertically transmitted symbiont, highly dependent on the selected vector due to its intracellular life cycle. This relationship with the vector may be the key factor driving the molecular variation in the *R*. *africae* genome as observed in the individual gene trees, where *R. africae* from different tick species clustered separately in some cases. Thus, a potential explanation for the formation of discrete clades in the individual gene trees could be attributed to vector–pathogen symbiosis. Indeed, Thu, et al. [72] noted a strong association between rickettsial genotypes and their host tick species in Japan. For *R*. *africae,* evolutionary divergence may be occurring between vector species of ticks. 

Several studies have highlighted that tick-borne pathogens in the microbiome of the ticks interact with each other [73,74,75]. Macaluso, et al. [73] found that when *Dermacentor variabilis* ticks had a rickettsial infection, the transovarial transmission of a second *Rickettsia* species was inhibited. Ponnusamy, et al. [76] noted that the *Amblyomma americanum* microbiome is altered based on the geographical distribution of the ticks, which in turn affects the composition and diversity of rickettsial species present. Importantly, although not detected here, African *Amblyomma* spp. may harbour rickettsiae other than *R. africae* [77,78]. However, no studies have been specifically performed to determine how combinations of organisms such as different *Rickettsia* spp. in the tick microbiome contribute to each other’s evolution. 

The prevalence of *R*. *africae* in *Amblyomma* spp. ticks ranged from 11.7% in South Africa to 35.7% in Zambia. The literature on the prevalence of *R*. *africae* in southern Africa is fragmented and limited to small geographical areas. In Angola, the overall prevalence was 24.4% and was divided between *A*. *pomposum* (72.1%) and *A*. *variegatum* (12.5%). Previous studies reported a prevalence of 45.5% [31] and 100% [79], respectively; although the sample sizes were very small (eleven and six, respectively), and Palomar, et al. [79] grouped their *Amblyomma* ticks into two pools, of which both tested positive. Our prevalence is much lower than other studies in the area; however, this could be attributed to their much smaller sample size. In this study, we found an overall prevalence of 25.7% in Mozambique (including *Amblyomma* spp. ticks collected from both wildlife and livestock), 33.5% for *A*. *hebraeum,* and 20.1% for *A*. *variegatum* from livestock. Magaia, et al. [30] reported a prevalence of 80% in *A*. *hebraeum* and 67% in *A*. *variegatum* ticks in Mozambique, whereas Matsimbe, et al. [28] documented a prevalence of only 4.5% in *A*. *variegatum*. Our results are within the expected range, and it is important to consider methodological differences between studies in terms of gene targets for PCR and the use of conventional or quantitative assays. 

There are no previous records of *R*. *africae* sequences from *Amblyomma* spp. ticks collected from wildlife in southern Africa. South Africa is the most well-represented country in the literature regarding *R*. *africae* prevalence in livestock ticks from southern Africa, with the prevalence in vectors ranging from 11.8% to 62.2% [44,47,58,80,81]. Our prevalence of 11.4% is on the lower end of what is expected for the country. In Zambia, only *A*. *variegatum* ticks were collected and the overall prevalence of *R. africae* was 35.7%. Previous studies in Zambia recorded widely different infection rates of 36.8% [31], 80.0% [35] and 15.6% [34]. In these studies, the sample sizes for *Amblyomma* spp. ticks were relatively small (19, 30 and 46, respectively). The prevalence for Zambia in the current study is within the range of other studies from the same areas. One study in Zimbabwe recorded a prevalence of *R*. *africae* of 40.0% (64/166) in *A*. *hebraeum* and 0% (*n* = 9) in *A*. *variegatum* [29]. In our study, an overall prevalence of 28.7% was found, with a prevalence of 29.3% in *A*. *hebraeum* and 9.1% in *A*. *variegatum*. Thus, our prevalence estimate is much lower in *A*. *hebraeum* but higher in *A*. *variegatum* than what was reported previously, although we can only compare to this one study. Several factors such as seasonality, sampling timeframe, and sample size may have influenced this discrepancy between the observed *R. africae* prevalences in Zimbabwe.

Statistically significant differences were observed between sexes in *A. hebraeum* ticks collected from livestock in Mozambique, where males had an *R. africae* prevalence of 40% compared to females with a prevalence of 19.3%. The observed higher prevalence in these males could be attributed to their migratory habits. Observations made by Stachurski [27] noted that *A. variegatum* males migrate during the night from predilection sites and from hosts in the same kraal. This migratory behaviour alongside cofeeding could increase the chances of males having higher infection rates for *R. africae* compared to the females, assuming that vertical and transstadial transmission in the wild is not efficient in this species.

In Angola, *A. pomposum* was statistically more likely to be infected with *R*. *africae* than *A*. *variegatum*. *Amblyomma pomposum* was restricted to the central western regions in Angola, placing travellers and indigenous inhabitants in these areas more at risk of contracting ATBF. In the livestock of Mozambique, *A*. *hebraeum* was more likely to be infected with *R. africae* than was *A*. *variegatum*. No statistical differences in the infection rates of *R*. *africae* between *Amblyomma* spp. were observed in other countries. This suggests that in all other countries, *A*. *hebraeum* and *A*. *variegatum* have a similar likelihood of transmitting *R*. *africae,* given equal biting rates. This is not unexpected, since *A*. *hebraeum* and *A*. *variegatum* are both considered to be the main vectors of *R*. *africae* in Africa [13]. 

With the high *R*. *africae* prevalence observed in the collected *Amblyomma* ticks in each country and the aggressive behaviour of these ticks, it is interesting to observe that there are few to no reports of ATBF or TBF in the local populations in southern Africa. Silva-Ramos and Faccini-Martínez [82] conducted a systematic review of the *R*. *africae* published case reports in humans, and found 108 documented cases, of which eight cases were in indigenous individuals, and the remaining infections were in travellers to southern Africa. Of these 100 cases of travellers, 80% were European, with South Africa being the most visited country.

Cattle are regarded as the primary host for all the collected *Amblyomma* species [15,51], while *A*. *eburneum* has also been documented to prefer African buffalo (*Syncerus caffer*) [51]. The majority of the rural communities are reliant on subsistence livestock farming, where the younger portion of the population are used as cattle herders. Although the primary hosts of the *Amblyomma* spp. are regarded as livestock, *Amblyomma* spp. are documented to parasitise humans, placing those in direct contact with their livestock at greater risk to be preyed upon [83], and therefore to contract *R*. *africae*. We can speculate that in impoverished communities in Africa, where *R*. *africae* is endemic, locals are exposed to the pathogen at a young age and develop immunity. Later, in their adult years, if they are infected with *R*. *africae*, symptoms are generally mild for which no medical attention is sought. For cases where medical attention is sought, they are neither documented as case reports nor published as clinical cases. Ultimately, very little or no documentation of the *R*. *africae* prevalence in individuals from *R*. *africae*-endemic areas is available.

## 5. Conclusions

This is one of the largest studies on *R*. *africae* prevalence in southern Africa. The prevalence in this study ranged from 11.7% to 35.7%, highlighting the need for these areas to include ATBF as a differential diagnosis when inhabitants and travellers present with flu-like symptoms. Limited research has been conducted on *R*. *africae* for many African countries, leading to probable underreported cases of ATBF. This study is the first in southern Africa to obtain sequence of *R*. *africae* from *Amblyomma* spp. ticks collected from wildlife and make these data publicly available. More research should be conducted on *R*. *africae* from ticks feeding on wildlife to clarify the phylogenetic placing of these sequences.

## Figures and Tables

**Table 1:** Primers used for amplification of specific targeted regions.

Target Region	Primer Name	Annealing Temperature (°C)	Sequence (5′–3′)	Expected Fragment Length (bp)	Reference
*ompA*	Rr190.70F	51	ATG GCG AAT ATT TCT CCA AAA	632	Mazhetese, et al. [58]
	Rr190.701R		GTT CCG TTA ATG GCA GCA TCT		
*ompB*	ompBF	50	ACA TK*G TTA TAC ARA GTG Y*TA ATG C	444	Pillay and Mukaratirwa [47]
	ompBR		CCG TCA TTT CCA ATA ACT AAC TC		
*omp*	17kDaF	50	AAT GAG TTT TAT ACT TTA CAA AAT TCT AAA AAC CA	450	Pillay and Mukaratirwa [47]
	17kDaR		CAT TGT TCG TCA GGT TGG CG		
*gltA*	gltAF	50	ACC TAT ACT TAA AGC AAG TAT Y*GG T	1234	Pillay and Mukaratirwa [47]
	gltAR		TCT AGG TCT GCT GAT TTT TTG TTC A		

* Degenerate bases ensure binding to all *R*. *africae* strains. Keto (K) can accommodate guanine (G) or thymine (T), while pyrimidine (Y) can accommodate cytosine (C) or thymine (T).

## Data Availability

The original contributions presented in the study are included in the article/Supplementary Material, and further inquiries can be directed to the corresponding authors.

## Supplementary Materials

The following supporting information can be downloaded at: https://www.mdpi.com/article/10.3390/microorganisms12081663/s1, Table S1: GenBank accession numbers; Table S2: *Rickettsia africae* infection rates; Figures S1–S4: Individual gene phylogenetic trees (*ompA*, *ompB*, *omp*, *gltA*).
