# Supplementary material for: Infection Rates and Characterisation of Rickettsia africae (Rickettsiaceae) Detected in Amblyomma Species from Southern Africa
Source: Microorganisms. 2024 Aug 13;12(8):1663. doi: 10.3390/microorganisms12081663 (PMC11357051; doi:10.3390/microorganisms12081663)
Supplement: Supplementary file 1 [file microorganisms-12-01663-s001.zip › Supplementary Data S2.pdf]

Supplementary Figure S1-S4: Individual figures of the Bayesian inference phylogenetic analyses of *R. afircae* for ompA, ompB, omp and gltA genes

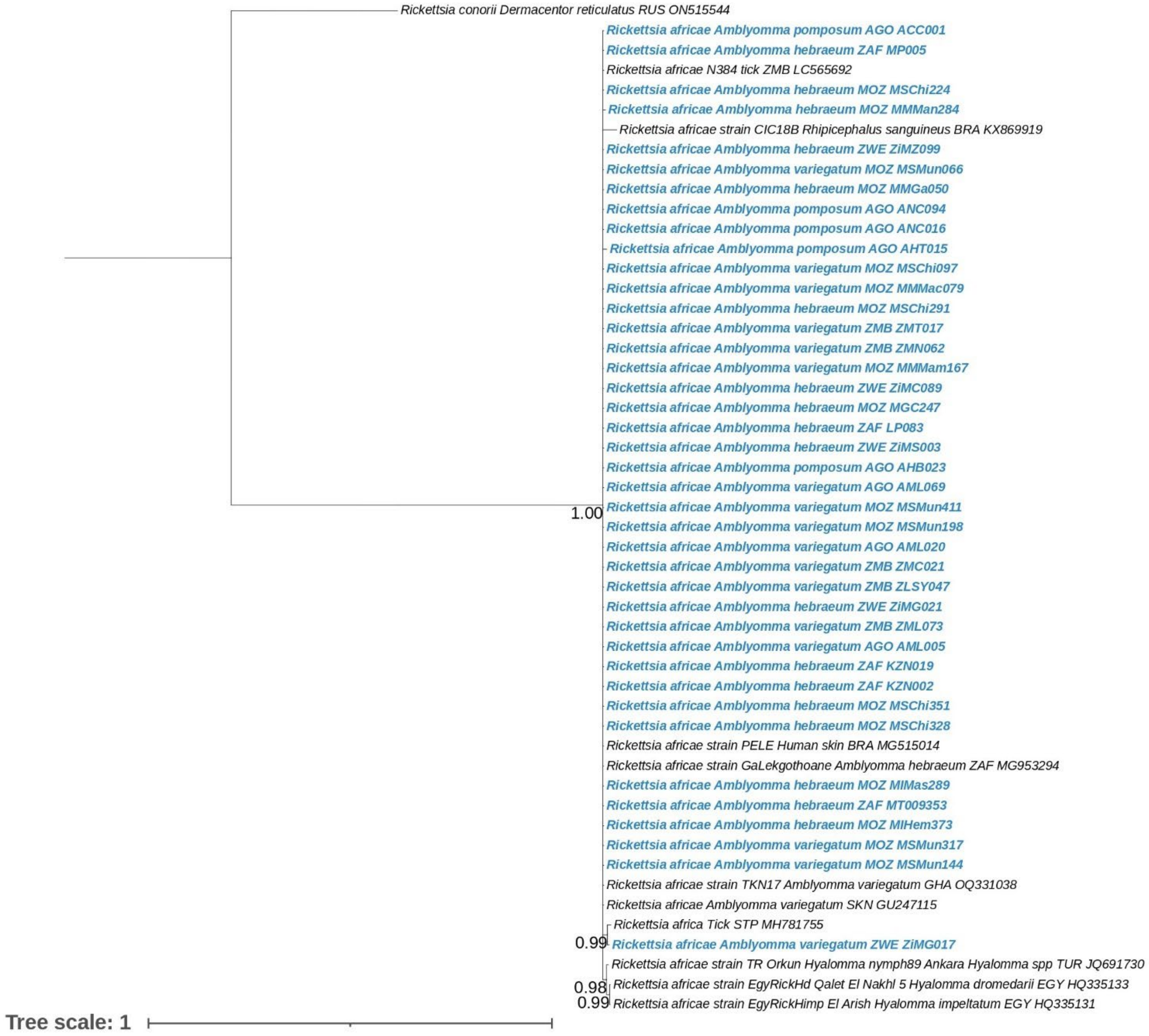

Figure S1: Bayesian inference analysis of the *ompA Rickettsia africae* gene. Analysis was done using the HKY model. Posterior probability is indicated at each branch node. Posterior probabilities < 0.85 have been removed. Sequences from this study are in blue. Country of origin is indicated in the sample ID as: AGO – Angola, BRA – Brazil, EGY - Egypt, GHA – Ghana, MOZ – Mozambique, RUS – Russia, SKN-Saint Kitts and Nevis, STP - Sao Tome and Principe, TUR – Turkey, ZAF – South Africa, ZMB – Zambia and ZWE – Zimbabwe.

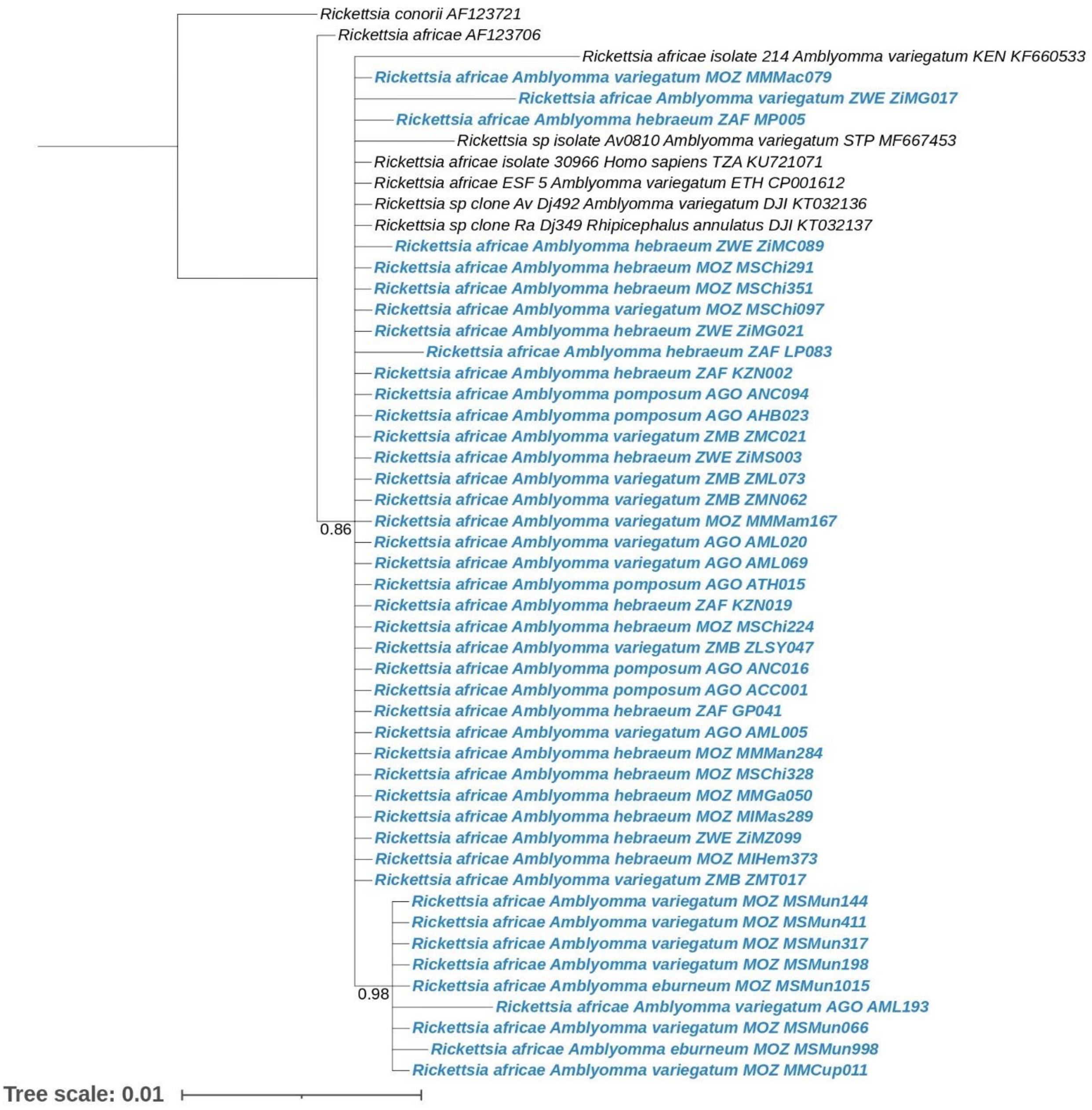

Figure S2: Bayesian inference analysis of the *ompB Rickettsia africae* gene. Analysis was done using the HKY model, with gamma rates. Posterior probability is indicated at each branch node. Posterior probabilities < 0.85 have been removed. Sequences from this study are in blue. Country of origin is indicated in the sample ID as: AGO – Angola, DJI - Djibouti, ETH – Ethiopia, KEN – Kenya, MOZ – Mozambique, STP - São Tomé and Príncipe, TZA – Tanzania, ZAF – South Africa, ZMB – Zambia and ZWE – Zimbabwe.

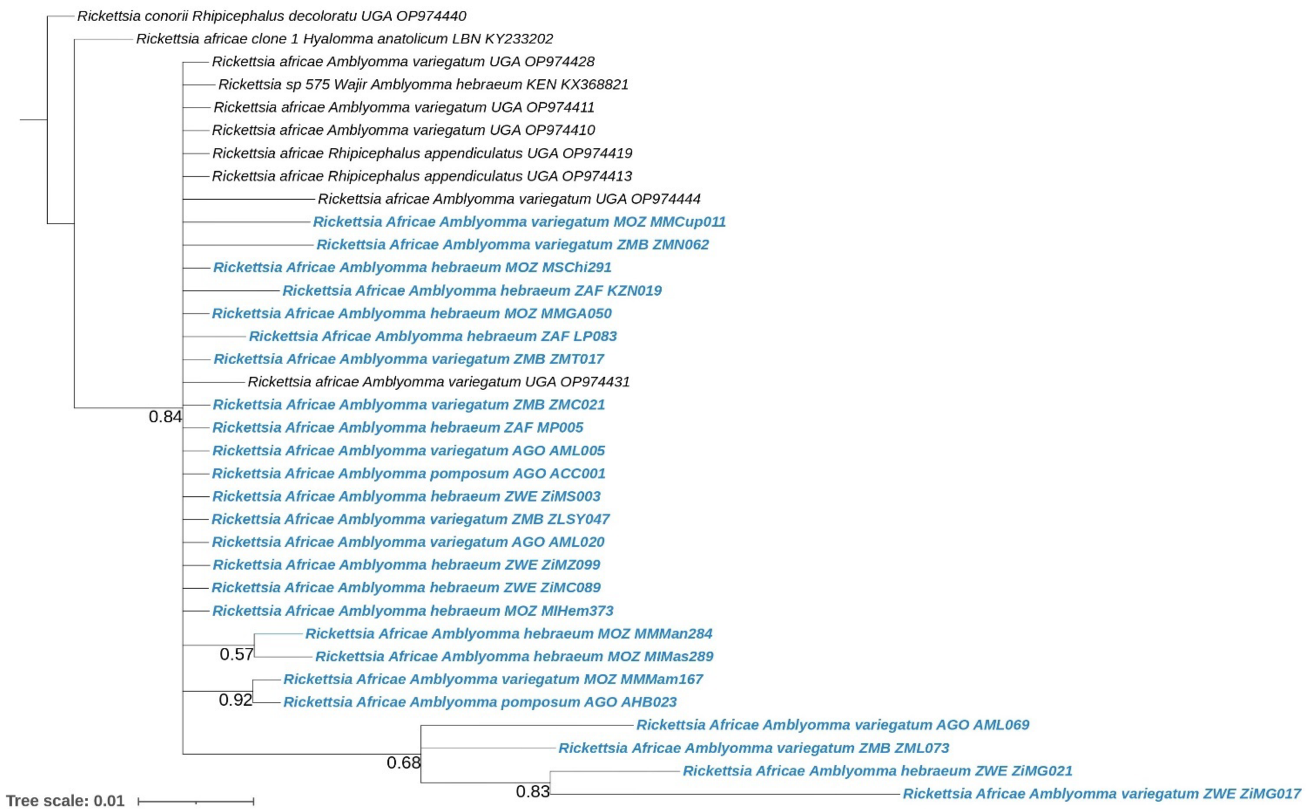

Figure S3: Bayesian inference analysis of the *omp Rickettsia africae* gene. Analysis was done using the HKY model. Posterior probability is indicated at each branch node. Posterior probabilities < 0.50 have been removed. Sequences from this study are in bold. Country of origin is indicated in the sample ID as: AGO – Angola, KEN – Kenya, KNY – Knysna, LBN - Lebanon, MOZ – Mozambique, ZAF – South Africa, UGA – Uganda, ZMB – Zambia and ZWE – Zimbabwe.

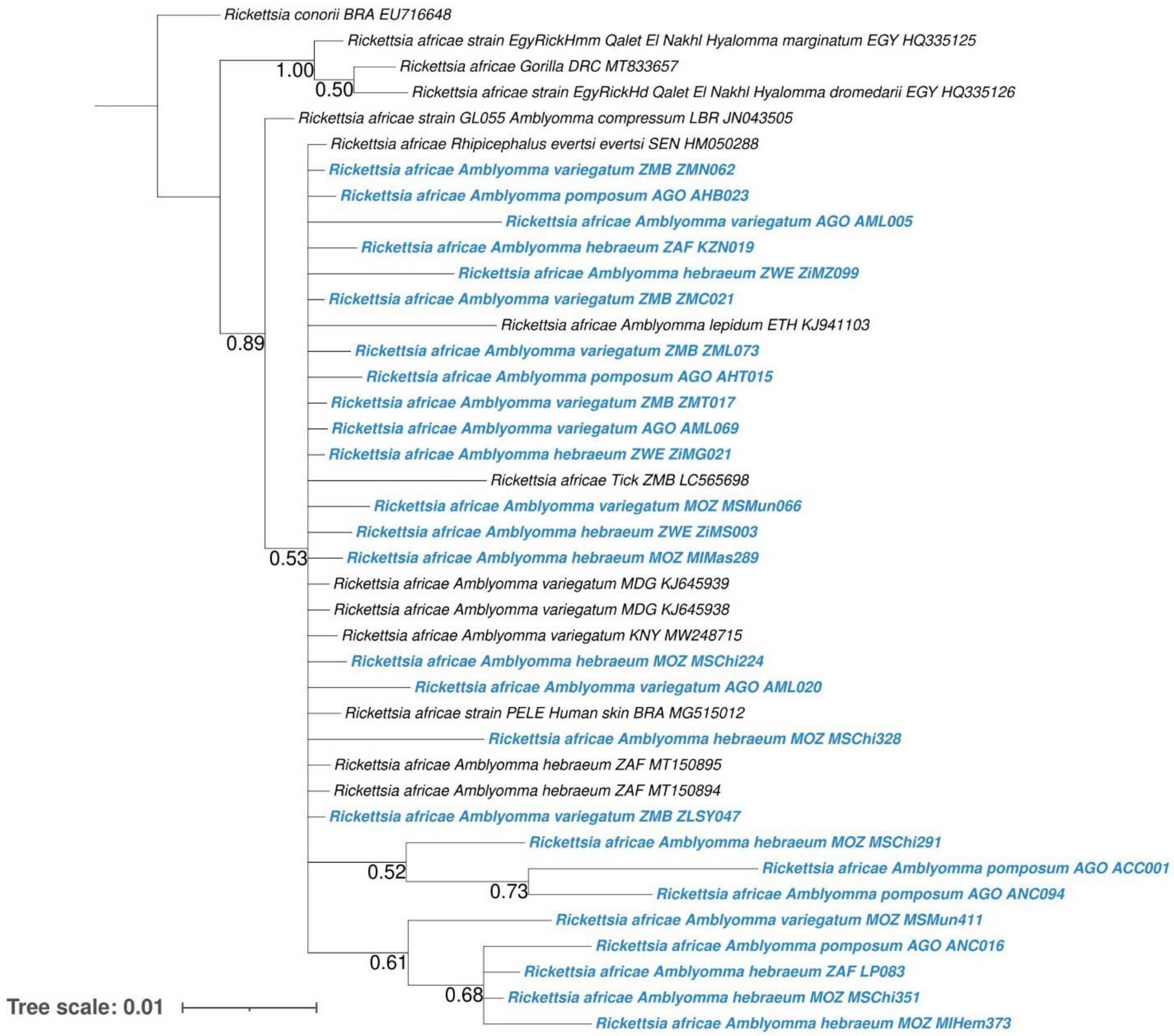

Figure S4: Bayesian inference analysis of the *gltA Rickettsia africae* gene. Analysis was done using the HKY model, with gamma rates. Posterior probability is indicated at each branch node. Posterior probabilities < 0.50 have been removed. Sequences from this study are in blue. Country of origin is indicated in the sample ID as: AGO – Angola, BRA – Brazil, DRC -Democratic Republic of the Congo, EGY - Egypt, ETH – Ethiopia, KEN – Kenya, KNY – Knysna, LBR - Liberia, MOZ – Mozambique, SEN – Senegal, ZAF – South Africa, ZMB – Zambia and ZWE – Zimbabwe
